# Temporal genetic structure in a poecilogonous polychaete: the interplay of developmental mode and environmental stochasticity

**DOI:** 10.1186/1471-2148-14-12

**Published:** 2014-01-22

**Authors:** Jenni E Kesäniemi, Marina Mustonen, Christoffer Boström, Benni W Hansen, K Emily Knott

**Affiliations:** 1 Department of Biological and Environmental Science, University of Jyväskylä, P.O. Box 35, Jyväskylä FI-40014, Finland; 2 Environmental and Marine Biology, Åbo Akademi University, Artillerigatan 6, Turku FI-20520, Finland; 3 Department of Environmental, Social and Spatial Change, Roskilde University, Universitetsvej 1, Roskilde DK-4000, Denmark

**Keywords:** *Pygospio elegans*, Poecilogony, Population genetics, Developmental mode, Genetic drift, Temporal, Sweepstakes reproductive success, Full-sibs

## Abstract

**Background:**

Temporal variation in the genetic structure of populations can be caused by multiple factors, including natural selection, stochastic environmental variation, migration, or genetic drift. In benthic marine species, the developmental mode of larvae may indicate a possibility for temporal genetic variation: species with dispersive planktonic larvae are expected to be more likely to show temporal genetic variation than species with benthic or brooded non-dispersive larvae, due to differences in larval mortality and dispersal ability. We examined temporal genetic structure in populations of *Pygospio elegans*, a poecilogonous polychaete with within-species variation in developmental mode. *P. elegans* produces either planktonic, benthic, or intermediate larvae, varying both among and within populations, providing a within-species test of the generality of a relationship between temporal genetic variation and larval developmental mode.

**Results:**

In contrast to our expectations, our microsatellite analyses of *P. elegans* revealed temporal genetic stability in the UK population with planktonic larvae, whereas there was variation indicative of drift in temporal samples of the populations from the Baltic Sea, which have predominantly benthic and intermediate larvae. We also detected temporal variation in relatedness within these populations. A large temporal shift in genetic structure was detected in a population from the Netherlands, having multiple developmental modes. This shift could have been caused by local extiction due to extreme environmental conditions and (re)colonization by planktonic larvae from neighboring populations.

**Conclusions:**

In our study of *P. elegans*, temporal genetic variation appears to be due to not only larval developmental mode, but also the stochastic environment of adults. Large temporal genetic shifts may be more likely in marine intertidal habitats (e.g. North Sea and Wadden Sea) which are more prone to environmental stochasticity than the sub-tidal Baltic habitats. Sub-tidal and/or brackish (less saline) habitats may support smaller *P. elegans* populations and these may be more susceptible to the effects of random genetic drift. Moreover, higher frequencies of asexual reproduction and the benthic larval developmental mode in these populations leads to higher relatedness and contributes to drift. Our results indicate that a general relationship between larval developmental mode and temporal genetic variation may not exist.

## Background

Several factors can affect the temporal stability of population genetic structure in the unpredictable marine environment. Unstable habitat can make a population more vulnerable to local extinctions and recolonizations [1,2]. Stochastic factors, such as variation in oceanic currents, can affect the movement of pelagic individuals and indirectly genetic patterns [3-5], or they can have a more direct effect, e.g. via high larval mortality. Especially in species with dispersive adults or larvae, temporal genetic differentiation could be caused by recruits migrating from different genetic sources at different times [6-10]. Sweepstakes reproductive success, extreme variation in the reproductive success of individuals, in which only a limited number of individuals contribute to the next generation, affects temporal population genetic structure in some species [11-14]. In small or fragmented populations, the enhanced effects of genetic drift also can lead to significant changes in temporal genetic structure [15-17]. Nevertheless, in most population genetic studies of marine invertebrates, only spatial patterns of genetic structure are examined without acknowledging the possibility for temporal genetic variation.

In many cases, the developmental mode of larvae can be used to generalize the dispersal potential of marine species (see [18] for review). Consequently, it may also indicate when spatial differentiation between populations and temporal variation within populations is likely [13,19]. Populations of species developing via dispersive pelagic larvae may be particularly prone to temporal genetic variation since such larvae are known to face high mortality in the plankton [20-22] and populations will receive recruits from potentially many different sources. On the other hand, species with direct development (those lacking a larval stage) and species with benthic, brooded, or encapsulated larvae that do not disperse long distances may be less prone to temporal genetic variation since such larvae are thought to be more protected from predation [20,23,24] and to show higher local recruitment. Lee and Boulding [13] studied temporal genetic variation in four closely related *Littorina* gastropod species with different larval developmental modes. They found the expected pattern of significant temporal genetic variation in two species with planktotrophic (pelagic) larvae, whereas the species with direct-developing offspring were temporally stable.

Even if a relationship between temporal stability of genetic structure and developmental mode exists, demographic differences between species and/or species-specific behaviours affecting larval recruitment could mask the general correlative patterns. Therefore, more data are needed before evaluating the generality of a relationship between temporal genetic variation and larval developmental mode. Poecilogonous species provide a means to examine this possible relationship without the influence of species-specific factors. Poecilogony refers to developmental mode polymorphism, in which there are multiple larval developmental modes within a single species [25,26]. Poecilogony is a rare phenomenon, known in some spionid polychaetes (e.g. [27,28]) and sacoglossan sea slugs [29]. In different poecilogonous species, individual females produce larvae developing via different developmental modes either simultaneously or seasonally, or the different modes are seen among multiple females either within or among populations (reviewed in [30]). Such variety implies that poecilogony could have arisen through different mechanisms in different species. These could include different genetic backgrounds, different environmental cues triggering the production of different larval types (developmental plasticity or bet-hedging), maternal effects, or a combination of these mechanisms (reviewed in [26,30]). For example, in *Alderia willowi*, poecilogony is based on reliable environmental cues, and females change the developmental mode of their larvae seasonally [29]. A polymorphic strategy may be favored in unpredictable habitats with spatial and temporal heterogeneity. In this case, poecilogony might be best described as a bet-hedging strategy, in which different phenotypes are produced in response to the unpredictability of the environment in an effort to maximize mean long term fitness [31,32]. Although poecilogony has not been proven to be a bet-hedging strategy (but see [30]), poecilogonous species are commonly found in intertidal habitats [25,27,28,33], which are characterized by rapid environmental fluctuations.

Our study species, *Pygospio elegans,* is a poecilogonous spionid polychaete [34,35] commonly found in a variety of sub-tidal and intertidal habitats [34,36,37]. This cold adapted species is widely distributed in the Northern hemisphere and has wide salinity tolerance [38]. *P. elegans* has been described as opportunistic [34,39] and can reach high densities, especially in nutrient rich intertidal mud and sand flats [40,41]. However in some regions (i.e. the northern Baltic Sea), relatively low densities are observed [37,42,43]. Across its distribution, populations are often described as patchy, and worm densities in populations have been observed to fluctuate over time ([34,40], pers. obs. JEK and KEK). Adult *P. elegans* worms inhabit sand tubes and are relatively sedentary. After fertilization via direct transfer of spermatophores from males to females, the females lay their embryos inside egg capsules within the maternal tube (up to 34 capsules in an egg string, [36]). Larval developmental mode varies and is related to the number of embryos laid per capsule [34,36,42,44]. When the number of embryos is large (>20/capsule), the larvae have a short brooding period and a long pelagic period. Larvae with this planktonic developmental mode actively swim and feed in the plankton. In laboratory experiments, the pelagic period of these larvae was 1–2 months, depending on the temperature [38]. When the number of embryos laid per capsule is small (1-2/capsule), the larvae are brooded throughout their development within the egg capsules, feeding on nutritional nurse eggs (adelphophagy) provided at egg-laying by the mother. Larvae with this benthic developmental mode lack a pelagic stage and build their own sand tube soon after release from the capsules. An intermediate developmental mode with an intermediate brooding period and a short pelagic period is also known [35,44]. In addition, *P. elegans* can reproduce asexually by fragmentation [45], but asexual reproduction has not been observed in all populations [42].

The developmental mode polymorphism observed in *P. elegans* makes the species particularly suited for examining the relationship between developmental mode and temporal population genetic structure. In some populations multiple larval types have been observed simultaneously or seasonally [35,36,42], but many populations of *P. elegans* are known to produce only one larval type (e.g.[34,40,46]). In Europe, a broad scale pattern in developmental mode and environment can be seen: the planktonic developmental mode is more common in populations from marine intertidal sand and mud flats of the North Sea, whereas populations with longer brooding (intermediate and benthic modes) are commonly found in the (estuarine) sub-tidal habitats of the Baltic Sea [42]. However, variation in developmental mode in this species does not appear to be a plastic response to variable salinity or temperature [46]. Nevertheless, environmental characteristics could serve as cues which trigger the levels of polymorphism in different populations. In the Baltic Sea*, P. elegans* is often seen associated with *Zostera marina* sea grass [42,43]. At local scales, these vegetated areas can have more stabilized sediment and show higher species richness and abundance of individuals due to the stabilizing effect of plant roots, reduced water movement and lower predation risk (e.g. [37,47-49]). Intertidal mud and sand flats however, are unstable habitats, e.g. due to disturbance from tidal flow, currents and human impact, which can lead to desiccation stress, sediment transportation and fluctuations in salinity, temperature and oxygen availability [39,50,51].

Since the planktonic, benthic and intermediate larval developmental modes of *P. elegans* differ in the number of larvae produced per female, the duration of brooding time within capsules and the duration of the planktonic period, as well as in the size of larvae at release from the capsules and the presence or absence of larval swimming setae, the larvae developing via these different modes are expected to differ in their dispersal potential and their susceptibility to predation. As a result, developmental mode of larvae may affect the population genetic structure of *P. elegans* both spatially [42] and temporally. Although somewhat higher population genetic connectivity is found among *P. elegans* populations with planktonic larvae, overall a pattern of significant spatial population genetic structure and low connectivity has been found among European *P. elegans* populations [42]. Moreover, asexual reproduction or mixed strategies of reproduction could affect population genetic structure [42,52,53].

In this study, we examined temporal genetic structure in *P. elegans* populations and its relationship to developmental mode. Following previous observations [13], we expected to find greater temporal genetic variation in the population with strictly planktonic larvae, whereas temporal genetic stability was expected for populations with predominately the benthic developmental mode. The possibility for greater temporal genetic variation in the planktonic population is expected to stem from the very high mortality rates known for the planktonic larvae of *P. elegans*[22] and higher gene flow among populations with planktonic larvae [42]. We also expected populations to differ in estimates of effective population size and sibship depending on developmental mode and the prevalence of asexual reproduction, with larger N_e_ and mostly unrelated individuals expected in those populations with primarily the planktonic developmental mode and lacking asexual reproduction. Our analysis of a poecilogonous species aims to clarify the connection between developmental mode and temporal population genetic variation and could shed light on the role of stochastic environmental variation on the evolution of marine invertebrate larvae.

## Methods

### Sample collection and DNA analyses

*P. elegans* adults were collected from seven sites in Europe during a period of four years (2008–2011; 2–3 temporal samples/site, Table 1). In a previous analysis, samples from these sites collected in 2010 showed significant genetic structure [42], so we considered each site to host a distinct *P. elegans* population. Sampling sites differed in terms of salinity (measured with portable refractometer with ATC), worm density and in the larval developmental modes that we observed (Table 1). The five sites sampled in the Baltic Sea were all sub-tidal and had sandy substrates, but differed in depth. In the brackish Finnish archipelago (Northern Baltic Sea), sediment sampling was done at two sites (FIA and FIF) by scuba diving in approximately 3–5 m deep water. In the Isefjord and Roskilde fjord estuary complex in Denmark (Zealand Island, Southern Baltic Sea), three sites were sampled by shoveling sediment from 50–100 cm deep water. Here, the sites also vary in exposure (DKR, located at the mouth of the estuary complex, is more exposed than DKV and DKH) as well as in salinity (Table 1).

**Table 1:** Sampling information

**Sea**	**Population**	**Code**	**Years sampled (number of genotyped individuals)**	**Salinity (psu)**	**Worm density**	Observed developmental modes*
Baltic Sea (st)	Ängsö	FIA	2008(53); 2009(52); 2010(42)	6-8	Low	B
	Fårö	FIF	2008(45); 2009(40); 2010(39)	6-8	Low	-
	Vellerup	DKV	2008(43); 2009(47); 2010(43)	20-21	Low to medium	I, P, B
	Rörvig	DKR	2009(42); 2010(40)	20-22	Medium	I, B, P
	Herslev	DKH	2008(24); 2010(42)	14	Low	I, B
Wadden Sea (it)	Netherlands	NET	2009(48); 2010(46); 2011(42)	28	High to very high	P, I, B
North Sea (it)	Drum sands	UK	2009(28); 2010(49)	28	High	P

*Pygospio elegans* sampling locations (st = sub-tidal, it = intertidal), population codes, and years sampled, with number of individuals genotyped per sample in parentheses. Salinity measured at each location, qualitative estimates of worm density and observed larval developmental modes are noted, with the most common developmental mode listed first.

*Observed larval developmental modes: B = benthic, I = intermediate, P = planktonic.

Marine intertidal sand and mud flats in the Wadden Sea (NET) and North Sea (UK) were also sampled. In both, sediment sampling was done during low tide when the sediment and worm tubes were partly exposed. In the Netherlands, we sampled a mudflat from the mainland side of Schiermonnikoog Island (site NET) and in the UK, the Drum Sands sand flat (near Edinburgh, Scotland) was sampled (Table 1). Even though we did not measure density of *P. elegans* during our collection, the difference between the intertidal marine sites and the sub-tidal Baltic Sea sites was striking. In UK and NET, the worm density was high with densely packed *P. elegans* tubes. In contrast, the distribution of worms was patchy and worm density was noticeably lower in the Baltic Sea. Densities from 150 to 2800 ind. m^2^ have been observed in the Northern Baltic Sea previously (pers. obs. CB), whereas 11 000 ind. m^2^ is common at the UK site [40,41].

For all populations, collecting was done at the same location each year by the same person/s. At each location, approximately 15 sediment samples were collected, with a minimum distance of 0.4 m and maximum distance of 20 m between any two samples. Fine scale genetic patterns may be likely in species with restricted larval dispersal (e.g. [53]), however it is not expected in *P. elegans* at this spatial scale (see [54]). The sediment samples were gently sieved with a 1 mm mesh sieve, and the worms’ sand tubes were removed with forceps and combined in a sampling bottle with sea water. In the laboratory, the tubes were placed in trays with sea water. After they emerged from their tubes, the worms were examined, sexed and checked for either sexual reproduction (indicated by the presence of gametes, which can be seen through the transparent body wall) or asexual reproduction (indicated by regeneration usually at both ends of the body, or multiple fragmented worm pieces within a tube). Sand tubes were also examined for the presence of egg capsules. When capsules were found, the developmental mode of the larvae was determined (Table 1). At site FIA we have observed only benthic larvae in egg capsules collected in early spring, but at site FIF we have not observed any sexually reproducing worms during our collections. All developmental modes have been observed in the Danish sites and NET, but benthic and intermediate modes predominate in the less saline environment in Denmark, whereas the planktonic developmental mode predominates in NET (Table 1). Only the planktonic developmental mode has been observed in UK [40,42]. Asexual reproduction in *P. elegans* was observed only in the Baltic Sea populations (Finland and Denmark) and was not frequent. These observations imply that developmental mode is associated with salinity, but because they are based on a limited number of samples taken primarily in spring, we cannot be sure that other developmental modes at these sites have not been overlooked. For example, we have visited the Finnish sites at different times during spring and summer, but have not observed gametes or larvae in the FIF individuals.

Adult worms were preserved individually in ethanol until DNA extraction. DNA was extracted using Qiagen chemicals and a Kingfisher magnetic processor (Thermo Fisher Scientific), after which the samples were genotyped using eight highly polymorphic microsatellite markers following protocols described in [54].

### Statistical analyses

Genetic variation within each sample was estimated by calculating expected and observed heterozygosity and gene diversity with Arlequin v.3.5.1.2 [55]. Also, allelic richness was calculated for each sample using a rarefaction method with HP-RARE [56]. Rarefaction standardizes the estimate to a minimum sample size to allow for comparison of the estimates among samples from the different populations. To investigate variation between temporal samples (within populations), the number of private alleles (alleles only seen in one sample) was caluculated for each population separately. Due to different sample sizes, the number of private alleles can only be compared among the temporal samples within a single population and not between populations. Deviations from Hardy-Weinberg equilibrium were calculated with Arlequin, and p-values were adjusted with Bonferroni correction. FSTAT [57] was used to estimate F_IS_ values for each sample and their statistical significance as well as to test for linkage disequilibrium between loci within the populations. Frequencies of null alleles were estimated using FreeNA [58].

To examine proportions of genetic variation explained by both spatial and temporal variation, analysis of molecular variance (AMOVA) was performed with two different sample sets using Arlequin (20 000 permutations). First, an analysis was carried out grouping the samples according to sample year (4 groups: 2008, 2009, 2010 and 2011). Second, the samples were grouped according to population (7 groups: temporal samples from the same population combined). To further investigate temporal genetic variation within the different populations, pair-wise F_ST_ values (using Arlequin and 10 000 permutations) and Jost’s [59] pairwise Dest values were calculated (using the R package DEMEtics, 3000 permutations [60]) among the temporal samples within each population (temporal F_ST_/Dest). Pair-wise comparisons of F_ST_ were also calculated among populations collected in the same year (2008, 2009, 2010). Linearized pair-wise F_ST_ values were analyzed with PCA in GenAlEx v. 6.5 [61] to visualize differences among the samples and populations.

After these analyses, we noticed a large temporal genetic shift in our samples from NET, as described in the results. To further investigate this shift, we used STRUCTURE v.2.3.4, [62] and also included some data reported in our previous spatial genetic analysis (populations from France and Netherlands, [42]) in order to identify potential source populations of the NET2011 sample. STRUCTURE was run assuming an admixture model and correlated allele frequencies. A prior with sampling locality information was used, and the analysis consisted of a burn-in of 250,000 generations followed by MCMC sampling of 400,000 generations. One to six possible clusters were tested, each with four replicate runs. The lowest likelihood scores were used to evaluate the number of genetic clusters in the data, but the result of interest was the placement of the NET2011 sample relative to the other samples. STRUCTURE results were visualized with the program Distruct 1.1 [63].

Since the benthic larvae of *P. elegans* are not expected to disperse after emerging from the maternal sand tube, we expected that populations with predominately benthic developmental modes would also have a large number of related individuals. To test this hypothesis, we did a sibship analysis using COLONY [64] to identify full-sib families within each temporal sample from the different populations. The analysis was run with no information on parental genotypes, and assuming both male and female polygamy as well as possible inbreeding. Following suggestions from Wang [65], the full-likelihood model was run with run length and precision set to medium. Data from seven of the microsatellite loci were used (excluding Pe12, the locus with the highest proportion of suspected null alleles) and genotyping error rate was estimated to be 0.0001 for each locus. Although *P. elegans* is not expected to live longer than one year in nature [38], its ability to reproduce asexually allows extension of the life-span of some individual genotypes. Consequently, we also analyzed population data sets in which the data from temporal samples were combined. We did not expect to find full-sib families consisting of individuals sampled in different years, but hypothesized that cross-year families would be more common in those populations with asexual reproduction (Baltic Sea). If full-sib pairs or families were found, one individual from each pair was chosen randomly and removed and the descriptive analyses (H_O_, H_E_ and F_IS_) and F_ST_ calculations were repeated to investigate the effect of related individuals on population genetic patterns. We also estimated mean relatedness (r) values for each sample using the maximum likelihood method in ML-Relate [66]. ML-Relate estimates relatedness for each individual pair-wise comparison, and the mean of these (excluding self-comparisons) was calculated for sample mean relatedness. Relatedness estimates were adjusted for the presence of null alleles.

If the same site is sampled at two or more time points, a short term effective population size (N_e_) can be calculated using temporal methods. These methods are based on changes in allele frequencies between the temporal samples [67]. Most methods used for calculating effective population sizes are based on assumptions of closed populations (no gene flow), discrete generations and random mating [68]. Although our populations are technically not closed, they are genetically differentiated from each other and show high self-recruitment rates [42]. Therefore, we expected that the data would be appropriate for the methods assuming closed populations. We first used the likelihood based method (MLNE) from Wang and Whitlock [69] to estimate N_e_ assuming closed populations (no migration), but then we also estimated N_e_ assuming open populations (migration allowed). In these analyses, we defined potential source populations as all the sites reported in this study, except when NET was the focal population. In this case, samples from other locations in the Netherlands and France, reported previously [42], were used as potential sources of gene flow (see results and discussion for more information). In all of the MLNE analyses, different maximum N_e_ values were tested with no change in the results, and N_e_ max = 10000 was used in the final analyses.

Mean N_e_ values over the sampling period were also estimated using the Moment Based Temporal method (MBT, [67]) implemented in NeEstimator [70]. TempoFs [71] was used to estimate genetic drift (observed allele frequency change) between the temporal samples. This method should produce unbiased results even with highly variable microsatellite markers. Mean Fs’ (genetic drift corrected for sampling plan) over all loci was calculated for the sample intervals. Sample plan 2 was used, in which samples are collected fatally before reproduction. In all of these temporal methods, the number of generations between temporal samples is required. For the Finnish sites (FIA and FIF), we estimated one generation per year (pers. obs. JEK), whereas for all other populations, two generations per year were assumed [36,40]. Again, since the life span of *P. elegans* is probably short in nature (worms lived for approximately one year in the laboratory [38]), the adults sampled in consecutive years are assumed to not be from the same cohort.

## Results

### Genetic diversity

Genetic variation in the temporal samples is summarized in Table 2. Genetic diversity was relatively high in most populations, lowest in Finland (FIF, H_E_ from 0.614 to 0.655) and highest in the UK (H_E_ from 0.797 to 0.799). There were fluctuations in allelic richness and heterozygosity among samples within most populations, with UK, DKR and FIF showing the most consistent values among samples (Table 2). Fluctuations in diversity did not follow any particular pattern. For example, in DKV diversity (H_E_, gene diversity, allelic richness) was lower in 2010 compared to the previous years, but in DKH diversity was higher in 2010 than in previous years. Most fluctuations in genetic diversity within populations were not of high magnitude, except in DKV, where there was a decline in variation from 2008 to 2010, and in NET, where there was a noticeable increase in variation in the 2011 sample. The fluctuations among samples within a population are indicated best by the numbers of private alleles (alleles observed only in one temporal sample) which were calculated for each population separately and, unlike allelic richness, are not comparable among populations but only among temporal samples within a population. Most extreme increases in the number of private alleles were seen in DKH from 2008 to 2010 (3.5 to 5.3), and in NET from the 2009 and 2010 samples (2.0 and 2.3, respectively) to the 2011 sample (4.4, Table 2). Overall, allelic richness was highest in UK, the population with only planktonic larvae, in line with our findings from a previous study of additional populations from a broader geographic scale [42].

**Table 2:** Genetic diversity and sibship patterns in the temporal samples

**Sample**	**H**_**O**_	**H**_**E**_	**GD**	**A**_**R (N = 40)**_	**PrivateA**_**R **_**within site**	**F**_**IS**_	**FS families (prob >0.98)**
FIA2008	0.583	0.660	0.600	10.8	1.9 *(N = 70)*	*0.116*	1
FIA2009	0.570	0.664	0.653	10.7	1.3	*0.140*	1
FIA2010	0.688	0.714	0.680	11.5	2.1	0.015	0
							All: 10
FIF2008	0.531	0.643	0.570	11.3	1.6 *(N = 58)*	*0.153*	0
FIF2009	0.478	0.614	0.558	10.0	1.5	*0.202*	0
FIF2010	0.567	0.655	0.588	11.0	1.8	*0.128*	0
							All: 1 (no cross-year)
DKR2009	0.584	0.696	0.644	10.3	3.7 *(N = 56)*	*0.146*	2*
DKR2010	0.606	0.698	0.622	10.6	4.2	*0.092*	1
							All: 5*
DKV2008	0.650	0.771	0.700	12.7	3.3 *(N = 74)*	*0.126*	4
DKV2009	0.704	0.775	0.721	12.2	2.2	*0.076*	2*
DKV2010	0.689	0.729	0.678	10.8	1.4	0.030	1
							All: 9*
DKH2008	0.596	0.677	0.596	10.0	3.5 *(N = 40)*	0.099	0
DKH2010	0.565	0.718	0.653	11.5	5.3	*0.189*	3
							All: 4 (no cross-year)
NET2009	0.586	0.703	0.616	11.4	2.3 *(N = 72)*	*0.169*	1
NET2010	0.627	0.717	0.669	11.4	2.0	*0.124*	4
NET2011	0.615	0.774	0.696	13.2	4.4	*0.182*	0
							All: 4 (no cross-year)
UK2009	0.606	0.799	0.765	13.2	4.4 *(N = 52)*	*0.242*	0
UK2010	0.664	0.797	0.738	13.7	5.3	*0.165*	0
							All: 1 (no cross-year)

Observed (H_O_) and expected (H_E_) heterozygosity, gene diversity (GD) and allelic richness (A_R_) based on a sample size of 40 for each sample (here, N = number of genes used in the rarefaction method of HP-rare). Private allele richness (PrivateA_R_) was calculated for temporal samples of each population separately (here, N [in italics] varies among populations, making the values comparable only among temporal samples within a population). Inbreeding coefficients (F_IS_, with significant values in italics) were calculated for each temporal sample. Number of full-sib families (FS) estimated within each temporal sample and within the combined dataset for each population (all) are listed. Samples marked with *have families consisting of more than two members (see text). In the combined data sets, cross-year full-sib families were found unless indicated.

Heterozygote deficiencies were seen in most samples and significant deviations from Hardy-Weinberg equilibrium were observed in some loci in most populations as reflected by significant positive F_IS_ values (Table 2). There were temporal changes in F_IS_ values in many populations. As shown in previous analyses [42,54], null alleles were estimated to occur in either low (<0.05) or moderate frequencies (<0.20) in most loci in some of the samples (including locus Pe7 in FIF, DKH and NET, locus Pe17 in DKV, DKR, DKH, NET and UK, locus Pe18 in DKH, NET and UK, locus Pe13 in FIA, FIF, NET and UK, and Pe19 in NET). Loci Pe15 and Pe12 were the most problematic, showing moderate null allele frequencies in almost all temporal samples. However, since the global F_ST_ and the F_ST_ values for each locus (data not shown) were similar when calculated with (ENA) and without estimating a null allele correction (F_ST_ = 0.0306, F_ST_ENA = 0.0308), we feel confident that the presence of null alleles has not affected our calculations of genetic structure. Regardless, Pe12 was eliminated from the data set when estimating the number of full-sib families with COLONY and when estimating the effective population size. Significant linkage disequilibrium was observed in only a few comparisons within some of the Danish populations (Pe7xPe15 in DKH & DKV, Pe7xPe17 in DKV, Pe7xPe13 in DKR, DKV & DKH, and Pe13xPe12 in DKR, Pe7xPe12 in DKV), occurring within some years, but not others. Although asexual reproduction is possible in *P. elegans,* we observed only two instances of identical multilocus genotypes in our samples: one pair of identical individuals in the DKV2008 sample and one pair of individuals in the DKR2010 sample. One individual from each of these pairs was removed from the COLONY, STRUCTURE and PCA analyses.

### Temporal and spatial genetic structure

AMOVA analyses (Table 3) indicated that the variation among the different populations was significant (3.61% of variation explained), and that this population structure was similar in different years (AMOVA grouping samples by year showed non-significant variation among years). Similarly, global F_ST_ values in different years were not significantly different (2008: 0.034, 2009: 0.046, 2010: 0.029, P = 0.617). Genetic structure between populations was also evident in our analysis of (within year) population pair-wise F_ST_ but there was temporal variation in structure among the Baltic Sea populations (Additional file 1). For example, in Denmark, most population pair-wise comparisons within a year were significant, however in 2010, DKV and DKH have non-significant pair-wise F_ST_. In Finland, the pair-wise F_ST_ values between FIA and FIF are low each year (F_ST_ < 0.01), and significant in 2008 and 2010, but not in 2009 (Additional file 1).

**Table 3:** **AMOVA results of spatial and temporal variation in *Pygospio elegans***

**Temporal groups (samples grouped by year)**				
Source of variation	Sum of squares	Variance components	Percentage of variation	Fixation indices (P-value)
Among years	22.3	−0.006	−0.27	F_CT_ = −0.003 (0.816)
Among populations within years	132.2	0.085	3.61	F_SC_ = 0.036 (<0.001)
Within samples	3436.3	2.273	96.66	F_ST_ = 0.033 (<0.001)
Total	3590.8	2.351		
**Geographical groups (samples grouped by population)**				
Source of variation	Sum of squares	Variance components	Percentage of variation	Fixation indices (P-value)
Among populations	110.9	0.067	2.85	F_CT_ = 0.029 (<0.001)
Among temporal samples within populations	43.6	0.020	0.85	F_SC_ = 0.009 (<0.001)
Within samples	3436.3	2.273	96.3	F_ST_ = 0.037 (<0.001)
Total	3590.8	2.360		

Temporal groups consist of samples from different populations grouped by sampling year (4 groups: 2008, 2009, 2010, 2011) and spatial groups consist of temporal samples grouped according to population (7 groups).

When the temporal samples were grouped according to population in AMOVA (Table 3), we found not only significant among population (spatial) variation (2.85% of variation explained), but also low but significant temporal variation within populations (0.85%). To clarify how specific temporal samples differed, we examined the samples from each population separately in pair-wise comparisons of F_ST_ and Dest (Table 4). In these analyses, significant differences were found between temporal samples in some but not all populations, and the F_ST_ and Dest values were generally low. There were no differences in temporal samples from UK and DKH. When significant differences between temporal samples were indicated, these were found between samples taken in subsequent years (2009 vs. 2010 in FIF, NET, DKR and DKV) as well as between samples from longer time periods (2008 vs. 2010 in FIA). Analysis of either F_ST_ or Dest indicated the same patterns of differentiation, except in DKR (Table 4). The largest differences among temporal samples was seen in the NET population, in which the 2011 sample was significantly differentiated from the two previous samples (2009 and 2010), which did not differ from each other (F_ST_ and Dest). Likewise, PCA analysis of linearized F_ST_ showed that in most cases, temporal samples from the same population clustered together (Additional file 2). However, NET2011 did not cluster with other temporal samples from NET and was placed between clusters of the Danish and UK samples, not grouping clearly with either (Additional file 2).

**Table 4:** **Temporal pair-wise F**_**ST**_**/Dest values for samples within each population**

**Finland**					
FIA	2008	2009	FIF	2008	2009
2009	0.002/0.018		2009	0.007/0.065	
2010	0.008*/0.055*	0.004/0.048	2010	0.004/0.040	0.011*/0.112**
**Denmark**					
DKR	2009		DKV	2008	2009
2010	0.008*/0.041		2009	0.002/0.006	
			2010	0.005/0.045	0.007*/0.061**
DKH	2008				
2010	0.007/0.062				
**Netherlands**					
NET	2009	2010	**UK**	2009	
2010	0.001/0.024		2010	−0.002/-0.001	
2011	0.038***/0.223***	0.031***/0.149***			

*P < 0.05, **P < 0.01, ***P < 0.001.

**Figure 1:**
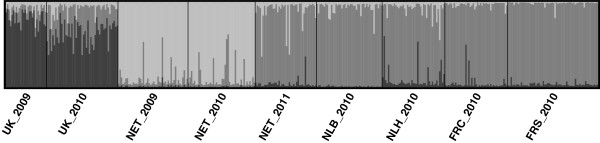
**Results of the STRUCTURE analysis used to clarify relationship of the NET2011 sample (K = 3).** Each column in the barplot represents an individual genotype with cluster assignment denoted by different colours. Note the clear difference in assignment between the NET2011 sample and previously sampled individuals at the same location. NET2011 clusters together with samples collected from two Dutch and two French populations sampled in 2010 (data from [42]) and is distinct from UK samples (this study).

We further investigated the differentiation of the NET2011 sample by performing additional pair-wise comparisons of F_ST_ and Dest with data from other nearby populations with planktonic larvae (from Netherlands and France) that had been sampled only in 2010 and were not the focus of this analysis (data from [42]). We found that although the NET2011 sample was significantly differentiated from earlier samples at the same site, it was not differentiated from other Dutch populations (NLH2010 and NLB2010) and French populations (FRS2010, and FRC2010 only in Dest) sampled in 2010 (Table 5). Moreover, we examined pair-wise F_ST_ and Dest between NET2011 and the 2010 samples collected from Denmark and UK (this study). Comparisons with the Danish samples indicated significant genetic differentiation, but with UK, the results were inconsistent (significant F_ST_, non-significant Dest) (Table 5). STRUCTURE showed clear assignment of NET2011 individuals to a different cluster than the other temporal samples from NET. Also, the analysis showed a shared cluster membership of NET2011 with the other Dutch and French populations sampled in 2010, which were distinct from the UK samples (K = 3 had the best likelihood, Figure 1).

**Table 5:** **Additional NET2011 F**_**ST**_**/Dest analyses**

	**NET2009**	**NET2010**	**NET2011**
NET2010	0.001/0.024		
NET2011	0.038***/0.223**	0.031***/0.149**	
NLB2010	0.053***/0.267**	0.047***/0.220**	**0.004/0.100**
NLH2010	0.050***/0.238**	0.048***/0.196**	**0.001/0.008**
FRC2010	0.039***/0.249**	0.040***/0.205**	0.009*/0.039
FRS2010	0.049***/0.251**	0.047***/0.232**	**0.004/0.032**
	**NET2011**		
DKR2010	0.036***/0.140**		
DKV2010	0.026***/0.104**		
DKH2010	0.025***/0.099*		
UK2010	0.020***/0.051		

*P < 0.05, **P < 0.01, ***P < 0.001.

Pair-wise F_ST_/Dest values comparing the NET2011 sample (in bold) to previous temporal samples at the same site, as well as samples collected from two Dutch (NLB2010 & NLH2010) and two French (FRC2010 & FRS2010) populations in the previous year (data from [42]) and to four 2010 samples from this study (DKR2010; DKV2010; DKH2010; UK2010).

### Sibship analysis and relatedness

Full-sibs were found within almost all temporal samples, but not in FIF and UK (Table 2). In most samples, full-sibs were pairs of individuals, but larger families were found in two Danish populations. In DKR2009 and DKV2009 samples, full-sib families of five and three individuals, respectively, were identified. In NET, full-sib pairs were seen in 2009 (one pair) and 2010 (four pairs), but none were observed in the 2011 sample. When temporal samples within a population were combined to identify cross-year full-sibs, typically the same full-sib families were identified as in the analyses of each temporal sample separately, but also additional full-sib families were seen. However, cross-year full-sibs (full-sib pairs or families consisting of individuals from different temporal samples) were found only in three Baltic Sea populations (FIA, DKR and DKV). In the analyses of DKR and DKV, in addition to full-sib pairs, two larger cross-year full-sib families were identified (DKR: families of five and four individuals, DKV: families of three and four individuals). Even though no full-sib families were identified in the temporal samples in FIF and UK when they were analyzed separately, in each of these populations one full-sib pair was identified when the temporal samples were combined. In both cases, the full-sibs identified were individulas within a single temporal sample (no cross-year full-sibs), indicating the effect of sample size on identifying potentially highly related individuals.

**Figure 2:**
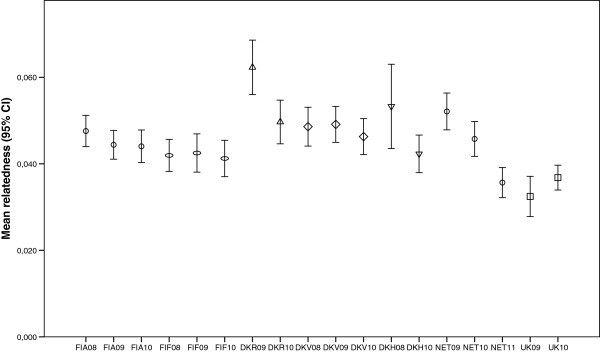
**Mean relatedness of individuals in the temporal samples.** Relatedness estimates (with 95% confidence intervals) were calculated in ML-Relate taking potential null alleles into account. Population codes and sampling years are described in Table 1. Symbols are used to separate populations (same symbols are used for temporal samples within a population).

Mean relatedness values for each sample are shown in Figure 2. In Finland and UK, the values were relatively stable among temporal samples, and the largest fluctuations were seen in two Danish sites (DKR & DKH) and in Netherlands. In DKR, the relatedness was significantly higher in 2009 compared to the 2010 sample (Mann–Whitney *U* test P = 0.031). In NET, the 2011 sample relatedness is significantly lower than in the previous years (NET 2010 & NET 2011: Mann–Whitney *U*-test P = 0.014; NET 2009 & NET 2011: P = 0.002), while 2009 and 2010 values were similar (Mann–Whitney *U*-test P = 0.432). In other sites, the relatedness values among temporal samples (pair-wise tests) were not significantly different.

### Effective population size

The different methods used for estimating contemporary N_e_ produced somewhat different results (Table 6): MLNE estimation assuming no gene flow resulted in the highest estimates, whereas the same method allowing migration (MLNE_open_) gave the lowest estimates. Overall, the values were low in comparison to estimations of population census sizes based on the densities of *P. elegans* in our sampling sites. Also, the 95% confidence intervals were wide and overlapping estimates for the different populations. With MLNE (without migration) the N_e_ estimations were similar in most sites (ranging from 142.1 to 355.5) except in UK, where a higher N_e_ was estimated (1117.8). With the Moment based temporal method (MBT), there was more variation among the estimates for the Baltic Sea populations, but the confidence intervals were overlapping, so the differences in estimated N_e_ are not significant. However, when comparing NET and the UK, the UK site has significantly higher N_e_ when estimated by MBT and MNLE_open_ methods. The overall trend seen in data is that the N_e_ estimates were highest in the strictly planktonic population, UK, and lowest in NET.

**Table 6:** Effective population size and genetic drift in the samples

	**MLNE (95% CI)**	**MBT (95% CI)**	**MLNEopen (95% CI)**	**Mean Fs’ (SE) between temporal samples**	
FIA 08-10				FIA 08-09	0.0036 (0.0043)
	217.4 (107–2894)	144.9 (68–721)	63.3 (47–93)	FIA 09-10	0.0110 (0.0023)
FIF 08-10				FIF 08-09	0.0147 (0.0066)
	186.2 (97–1077)	142.6 (62–1654)	42.7 (32–59)	FIF 09-10	0.0202 (0.0071)
DKV 08-10				DKV 08-09	0.0105 (0.0105)
	176.8 (118–314)	169.0 (81–715)	52.2 (39–72)	DKV 09-10	0.0166 (0.0056)
DKR 09-10					
	213.3 (97–2989)	80.6 (60–267)	42.5 (31–64)	DKR 09-10	0.0187 (0.0116)
DKH 08-10					
	355.5 (134-inf)	82.8 (45–201)	45.5 (29–73)	DKH 08-10	0.0166 (0.0093)
NET 09-11				NET 09-10	0.0077 (0.0301)
	142.1(103–208)	58.0 (37–94)	43.7 (34–57)	NET 10-11	0.0635 (0.0297)
UK 09-10					
	1117.8 (170-inf)	660.6 (109-inf)	89.7 (57–168)	UK 09-10	0.0005 (0.0041)

Estimates of effective population size (N_e_) and their 95% confidence intervals (inf = infinite) calculated with different methods (see text for more information). Mean Fs’ (SE = standard error) is the observed allele frequency change (i.e. drift) between the temporal samples (from analysis with TempoFs).

The estimated temporal shifts in allele frequencies (i.e. drift) between the samples were quite high in the Baltic Sea populations (Fs’ from 0.0036 to 0.0202, Table 6). In the UK, Fs’ was low (0.0005), allowing higher N_e_ estimations. The largest change in allele frequencies was seen between the NET2010 and 2011 samples (Fs’ = 0.0635), and this incidence of strong genetic drift is likely the cause of the low mean N_e_ values estimated for the NET site over the whole sampling period. In contrast, the genetic drift estimate for NET2009 and NET2010 was an order of magnitude lower (Fs’ = 0.0077).

### Analyses with full-sibs removed

To reveal the effect of full-sibs within a sample on our analyses, one individual of each full-sib pair was randomly removed (in case of larger families, all but one individual was removed) and some of the analyses were repeated with the reduced dataset (see Table 2 for samples with full-sibs). As mentioned previously, in the samples with identical individuals (one pair in DKV2008 and in DKR2010), one individual from each pair was removed in the analyses. The removal of close relatives did not have a large effect on the observed and expected heterozygosities, although in DKR2010 and DKV2009 F_IS_ values were no longer significant (Additional file 3). Removal of full-sibs did not affect the estimates of effective population size (results not shown). However, the removal of full-sib individuals had significant effects on temporal population genetic patterns in some populations (Additional file 4). In FIA and DKH, the F_ST_ significance patterns did not change (significant genetic differentiation between FIA2008 & FIA2010 remained). However in DKR and DKV, the significant F_ST_ comparisons observed with full data set were not observed when full-sibs were removed. In the Netherlands, the NET2011 still remained significantly differentiated from the earlier temporal samples, indicating a strong genetic shift.

As expected, the removal of full-sib individuals also decreased the mean relatedness values calculated for each sample (using ML-Relate, see Additional file 5). For example, without full-sibs the mean relatedness decreased from 0.0623 to 0.0499 in the DKR2009 sample, and the differences seen in relatedness values between the temporal samples of DKR were no longer significant (Mann–Whitney *U* test P = 0.639). In NET, however, relatedness in the 2011 sample still remained significantly lower than relatedness in previous years (NET 2010 & NET 2011: P = 0.013; NET 2009 & NET 2011: P = 0.001; NET 2009 & NET 2010: P = 0.429).

## Discussion

Life history traits associated with the planktonic development of marine invertebrate larvae, including high fecundity, high larval mortality, and, for broadcast spawning species, low fertilization success, have been hypothesized to contribute to temporal dynamics in populations and their genetic structure [13,19,21,33]. Indeed, temporal variation in genetic structure has been described for some marine species, and stochastic effects on larval life stages are often cited as the potential causes of this variation [11-13,72-75]. Adaptations such as internal fertilization and parental protection may lead to increased survival of larvae [76] and reduce the role of stochastic factors on temporal genetic variation [13]. A comparison of closely related snails in the genus *Littorina* with different larval developmental modes showed that species with direct-development (hatching as juveniles) had more temporal stability in genetic variation, due to their lower fecundity and lower offspring mortality, whereas species with planktotrophic (pelagic) larvae showed temporal fluctuations in genetic structure, possibly due to sweepstakes reproductive success [13]. We examined the temporal population genetic structure in *P. elegans*, a poecilogonous species in which the developmental mode of larvae is known to vary among and within populations [42]. Our study adds to the relatively few studies investigating temporal genetic variation in marine invertebrate species. In addition, to our knowledge, this is the first such study done with a poecilogonous species, which provides a within-species examination of the proposed relationship between developmental mode and temporally varying genetic structure.

We expected to find temporal variation in the genetic structure of *P. elegans* populations in which planktonic larvae are predominant: UK, having only planktonic larvae; and NET, where planktonic larvae predominate, even though other developmental modes are also observed in this population. Both of these sites are intertidal habitats with high worm density. Planktonic *P. elegans* larvae are known to suffer very high mortality [22], which could lead to temporal variation in genetic structure. The high and fluctuating density of worms in these two populations ([40], pers. obs. JEK, KEK) could also reflect the opportunistic behavior of the worms (extinction and re-colonization are possible) or a high influx of recruits, both of which could lead to increased temporal genetic variation. Fecundity is also expected to be higher in females producing planktonic larvae compared to those producing benthic larvae, as in other poecilogonous polychaetes (*Strepblospio benedicti*, [77]*;* and *Boccardia proboscidea*, [28]). On the other hand, we expected to find temporal stability in the genetic structure of *P. elegans* in populations from the Baltic Sea (Finland and Denmark), where benthic and intermediate larvae predominate. Although mortality rates are not known for benthic larvae of *P. elegans*, we assume that they are significantly lower than those for planktonic larvae. In contrast to our expectations, we found temporal genetic variation in populations from the Baltic Sea, temporal genetic stability in the UK population, and evidence of a genetic turnover in NET. Temporal variation in mean relatedness within populations was also found. Several possible explanations for our findings are discussed below.

The most significant change in genetic structure that we observed occurred in NET between years 2010 and 2011. This may be an example of a population turnover in response to extreme environmental conditions. *P. elegans* is known to colonize new or defaunated areas relatively quickly [46,78,79], and this could allow for the revival of an existing *P. elegans* population after a crash in population density. The NET sampling site was muddier in 2011, noticeably different than in the previous years, and the *P. elegans* distribution at this site was also patchier and less dense than previously (but still very high in comparison to populations in the Baltic Sea). It is possible that the harsh winter of 2010/2011 decreased *P. elegans* density at this site, which subsequently increased with immigration of planktonic larvae from nearby populations. Indeed, we saw that the NET2011 sample is genetically differentiated from temporal samples collected earlier at the same site (NET2010 & NET2009), but undifferentiated from samples from e.g. other Dutch populations collected in 2010 (Figure 1, Table 5). Allelic variation (allelic richness & number of private alleles) was higher in the NET2011 sample compared to the previous years (Table 2), even though a decrease in diversity is usually expected after a population turnover ([1,80], but see also [9]). Therefore, it is likely that the NET site was recolonized either by a genetically diverse pool of larvae or by individuals from several different sources. This conclusion is also supported by the lower mean relatedness estimate for the NET2011 sample compared to samples from the same site in previous years (Figure 2). Harsh weather conditions (e.g. severely cold winters) are known to affect benthic community structure in marine species [81,82]. For example, Naumov [83] reported that even though sediment covered by ice and melting ice plates can remove individuals and lead to high mortality of benthic species in intertidal mudflats, these communities make a fast recovery.

Like NET, the sand flat inhabiting population in UK may also be subject to extreme environmental variation. Although we did not observe temporal variation in population structure in UK, our sampling scale may not have been sufficient to observe temporal dynamics in this population with planktonic larvae. We had only two temporal samples for the UK population (2009 and 2010), covering two generations of *P. elegans*. A study performed over a longer time scale might reveal patterns resembling metapopulation dynamics in this species with local extinctions/population crashes and recolonizations with migration connecting local populations (see [84]). Metapopulation dynamics may be more likely in *P. elegans* populations in the North Sea and Wadden Sea, where the high probability of physical disturbance in the intertidal mud and sand flat sites render the populations more vulnerable to crashes between high density peaks. However, despite density changes observed in these populations, regular population extinctions have not been reported for *P. elegans.* Local extinctions and recolonizations can be noticed with genetic analysis of temporal samples (similar to what was seen here for NET), but that is not always the case (e.g. [2]). Future studies with *P. elegans* would benefit from a more direct investigation of population dynamics, e.g. investigating age structure within a population and examining genetic patterns and population assignments in cohorts of larvae despite the challenges of genotyping larvae or young juveniles.

Significant temporal genetic structure was found in most of the Baltic Sea populations, which had predominantly benthic or intermediate larvae and visibly lower worm densities. The F_ST_ and Dest values in the significant pair-wise comparisons among temporal samples in these populations were not of high magnitude, however, and were lower than seen in NET (Table 4). Commonly, the sub-tidal habitats of the Baltic Sea do not support high densities of *P. elegans*[37,42,43]. If low density indicates small effective population size, random genetic drift is most likely the primary factor influencing temporal genetic variation [15]. Drift has been suggested to cause the temporal genetic variation seen in another polychaete species with benthic, non-dispersive larvae [16]. Alternative explanations for the observed temporal variation in the Baltic populations, e.g. population turnovers or immigration from many source populations leading to changes in allele frequencies, are not expected to be likely, since the predominance of longer brooding in these populations decreases the chances of gene flow through larval dispersal. Supporting the hypothesis of self-recruitment, a previous study [42] showed that in *P. elegans* the genetic connectivity is higher among the North Sea populations with planktonic larvae, but local recruitment rates are also likely to be high in all populations. For example, even in the exposed sand flat population in UK, local currents may facilitate larval retention [40]. Continuous high local larval retention could lead to the spatial genetic structure pattern seen in *P. elegans* (shown here and in [42]), as well as to the high F_IS_ values observed in these populations. Recent studies have shown that local larval recruitment in the marine habitat may be more common than assumed [4,85,86].

However, after the removal of full-sib individuals from our dataset, previously significant pair-wise F_ST_ patterns seen in the Danish sites DKR and DKV were no longer significant, whereas in FIA and NET, the significant pattern remained. Therefore, the presence of full-sibs in the dataset may affect our interpretation of the genetic patterns (and see [87,88]). We identified full-sib families within temporal samples of most of the *P. elegans* populations. Given that at each location multiple separate sediment samples were collected, it is remarkable that potential full-sib families could be identified, and we think that the presence of full-sib families in the samples is not due to possible collection biases, but that it represents the natural state of the populations. Since full-sib families were found in the populations with primarily benthic and intermediate developmental modes, we expect that the relatively lower dispersal capability of these larvae facilitates local recruitment and and thus leads to increased relatedness within these populations. However, larval behavior, physical factors and oceanic currents may also entrain planktonic larvae to settle in their natal sites [89,90] and we cannot exclude this possibility for *P. elegans.*

Recently, Iacchei and colleagues [88] discussed how the presence of many closely related individuals within a population could be due to related larvae dispersing and settling together, sweepstakes reproductive success, or high self-recruitment. The idea of related larval pools dispersing together is somewhat controversial, but supporting evidence has come from studies which identified related individuals in cohorts of larval recruits [4,91-93] and from simulation studies [94,95]. The lack of full-sib families in UK and in NET2011 (after the genetic turnover), however, suggests that for populations of *P. elegans*, the prevalence of planktonic larvae reduces the likelihood of finding full-sibs in a sample. Although we did not examine larval cohorts to specifically test for sweepstakes reproductive success, our results also suggest that in *P. elegans* there is low variation in the reproductive success among individuals, since larger full-sibs families are more likely to be seen when variation in reproductive success is high [4,96]. Samples of additional populations with only planktonic larvae would be required before we can be certain about a possible connection between developmental mode and the presence of full-sib families in a population.

Asexual reproduction in *P. elegans* observed in the Baltic Sea populations may also have a role in explaining the observed patterns of relatedness, and can also explain why cross-year full-sib families were found in Finland and Denmark. Asexual reproduction likely allows certain genotypes of *P. elegans* to persist in a population over multiple years. There are no reported cases of asexual reproduction at the UK site (repeated collections done by Bolam [40] and pers. obs. JEK, KEK) and we have not observed asexual worms in Netherlands [42]. Although we used a strict probability limit to accept identification of a full-sib family, several factors could influence the analysis, including sample size, number of loci, and genotyping error rates [64,97,98]. The influence of sample size is already shown in our results: we found more full-sib families when analyzing the combined datasets (temporal samples combined per population) than when the temporal samples were analyzed separately (Table 2).

Our estimates of effective population sizes in *P. elegans* reflect our conclusions of a genetic turnover in NET and genetic drift in the Baltic Sea. Overall, our N_e_ estimates for the different populations were low, with overlapping confidence intervals. The high N_e_ estimates were for UK, the population with only planktonic larvae, which also had temporally stable genetic structure and highest genetic diversity. In general, higher N_e_ estimates are expected for species with planktonic larvae [13,21]. However, generalization of N_e_ and developmental mode may not be possible due to sweepstakes reproductive success [19,99,100]. For example, some recent studies have estimated low N_e_ for marine species with planktonic larvae (oyster: [91] and fish: [101,102]). For *P. elegans*, we do not expect sweepstakes reproductive success to have a major role, since species with internal fertilization and brooding are less prone to experience such extreme variation in reproductive success (see [19]), however, a direct test is needed.

It should be noted that the three methods used to estimate contemporary N_e_ from temporal allele frequency changes can be biased and result in low estimates if there are overlapping generations, small sample sizes, or short time intervals between samples [103,104]. Also, non-random mating, female biased sex ratio (which is known for *P. elegans*, [36], pers. obs. JEK), and HWE deviations may bias the estimates. In addition, MLNE estimations with migration (MLNE_open_) may be biased if all potential source populations are not sampled [69]. The N_e_ estimates obtained with this method were lower than those using MLNE and assuming closed populations, a pattern which has also been reported for other species [72]. Although we used different methods to estimate N_e_, all have limitations, and our study system may not be ideally suited to these analyses. The wide confidence intervals indicate that these estimates of N_e_ should be considered with caution. Although it is suggestive, we cannot say with certainty whether the low N_e_ estimated for the Baltic Sea populations is due to the occurrence of the benthic larval developmental mode, since other unexplored population demographic factors may play a role.

Our expectations for temporal genetic variation in the populations with planktonic larvae and temporal genetic stability in the populations with benthic larvae were based on assumptions that may be incorrect or oversimplified. We hypothesized that higher mortality of planktonic larvae, in addition to higher gene flow, would predispose *P. elegans* populations with planktonic larvae to greater temporal genetic variation. Theoretically, high mortality of larvae via predation, variance in successful larval dispersal and recruitment (e.g. via natural selection), or variance in reproductive success can lead to fluctuating genetic patterns (see [19]). However, our assumptions disregarded the potential role of also adult mortality. Environmental variables influence population dynamics through effects on both life stages, and in this study, the populations studied not only differed in developmental mode, but also in their habitat (subtidal vs. intertidal, muddy vs. sandy sediment), salinity, worm density, and may have also experienced different degrees of disturbance. Species that inhabit unstable environments, steep environmental gradients or marginal areas of their distribution are likely to (be stress tolerant or) show plasticity [105], or have adaptations to local conditions [106-108]. Therefore, it is not surprising to find that opportunistic species, including *P. elegans*, with short life span are commonly seen in intertidal habitats which have a high magnitude of disturbance [39,40,109]. The genetic turnover observed in NET provides an argument for the role of stochastic adult mortality as well as larval development mode (recolonization by planktonic larvae) in creating the observed temporal genetic variation. On a broad scale, variation in developmental mode in this species may be the result of an effective bet-hedging strategy in a species which can tolerate a wide-range of environments.

## Conclusions

In the marine environment, larval developmental mode can affect population dynamics and stability via its effects on fecundity, population growth rate and survivorship of larvae and juveniles. It has been suggested that species with planktonic larval developmental modes would show more fluctuations in abundance over time [21,24,33] and be prone to temporal genetic variation [13]. In our study of a poecilogonous polychaete species, temporal genetic variation appears to be not only due to larval developmental mode, but also to stochastic environmental effects on adults. Although temporal samples in populations with planktonic larvae were mostly genetically stable, we did find evidence for a genetic turnover in one population inhabiting an intertidal mudflat in Netherlands. Here, (re)colonization of the population by planktonic larvae from neighboring populations following an extreme environmental stress can explain the genetic turnover. We also found low but significant genetic differentiation among temporal samples from the Baltic Sea populations with predominantly benthic larval developmental mode. At least at the Finnish sites, these patterns are likely due to genetic drift, however in Denmark, it seems that the presence of closely related individuals created the observed significant temporal genetic differentiation. Sub-tidal and/or brackish (less saline) habitats may support smaller *P. elegans* populations, and together with higher frequencies of the benthic larval developmental mode and asexual reproduction, these may be more susceptible to the effects of random genetic drift and higher relatedness within sites. Our results indicate that a general relationship between larval developmental mode and temporal genetic variation may not exist (at least within species, but possibly also across species). In *P. elegans,* and potentially other benthic invertebrates, temporal genetic stability of a population is likely described by a complex interplay of larval developmental mode and environmental characteristics of the focal habitat.

### Availability of supporting data

The data set supporting the results of this article is available in the Dryad Digital Repository, DOI: http://doi.org/10.5061/dryad.r90j0.

## Competing interests

The authors declare that they have no competing interests.

## Authors’ contributions

JEK and KEK conceived the study. JEK, CB, BWH and KEK collected samples used for the study. JEK and MM collected all genetic data. JEK, MM and KEK analyzed the genetic data. JEK and KEK wrote the manuscript, incorporating contributions from all authors. All authors read and approved the final manuscript.

## Supplementary Material

Additional file 1 **Pair-wise F**_**ST **_**values for among populations comparisons within years.** These results show significant genetic structure among the populations: the few exceptions are among some geographically close populations (as described in the text). Click here for file

Additional file 2 **Results of PCA analysis on linearized pair-wise F**_**ST**_**.** Differences among the temporal samples and populations are visualized. Click here for file

Additional file 3 **Genetic variation in the temporal samples after the removal of some full-sib individuals.** Observed (H_O_) and expected (H_E_) heterozygosity and inbreeding coefficients (F_IS_) are reported. Click here for file

Additional file 4 **Within population temporal pair-wise F**_**ST **_**values after the removal of some full-sib individuals.** Click here for file

Additional file 5 **Mean relatedness (r) in the samples calculated with and without full-sibs (no FS).** In DKR 2009, the mean relatedness is significantly lower after the removal of full-sib individuals. Click here for file
