# Supplementary material for: Temporal genetic structure in a poecilogonous polychaete: the interplay of developmental mode and environmental stochasticity
Source: BMC Evol Biol. 2014 Jan 22;14:12. doi: 10.1186/1471-2148-14-12 (PMC3905951; doi:10.1186/1471-2148-14-12)
Supplement: Additional file 1 — Pair-wise FST values for among populations comparisons within years. These results show significant genetic structure among the populations: the few exceptions are among some geographically close populations (as described in the text). [file 1471-2148-14-12-S1.pdf]

Additional files

Additional file 1. Pair-wise F_{ST} values for among populations comparisons within years.

Pair-wise F_{ST} values for among populations comparisons within years. These results are similar to what was reported in Kesäniemi *et al*. 2012b and show strong structure among most populations except for some geographically close populations (as described in the text). Pair-wise comparisons on grey background are discussed in the results.

**2008**	FIA	FIF	DKV
FIA	0		
FIF	0.009*	0	
DKV	0.052***	0.043***	0
DKH	0.026***	0.026***	0.016**

**2009**	FIA	FIF	DKR	DKV	NET	UK
FIA	0					
FIF	0.010	0				
DKR	0.046***	0.055***	0			
DKV	0.048***	0.062***	0.023***	0		
NET	0.029***	0.050***	0.027***	0.019***	0	
UK	0.104***	0.125***	0.071***	0.045***	0.064***	0

**2010**	FIA	FIF	DKR	DKV	DKR	NET	UK
FIA	0						
FIF	0.008*	0					
DKR	0.019***	0.023**	0				
DKV	0.020***	0.025***	0.010*	0			
DKH	0.010**	0.018***	0.009*	0.006	0		
NET	0.018***	0.025***	0.020***	0.016**	0.011**	0	
UK	0.061***	0.074***	0.055***	0.046***	0.041***	0.051***	0

*P<0.05, **P<0.01, ***P<0.001
