# Supplementary material for: Temporal genetic structure in a poecilogonous polychaete: the interplay of developmental mode and environmental stochasticity
Source: BMC Evol Biol. 2014 Jan 22;14:12. doi: 10.1186/1471-2148-14-12 (PMC3905951; doi:10.1186/1471-2148-14-12)

Additional file 2. Results of PCA analysis on linearized pair-wise F_{ST}.

Results of PCA analysis on linearized pair-wise F_{ST}. Differences among the temporal samples and populations are visualized.

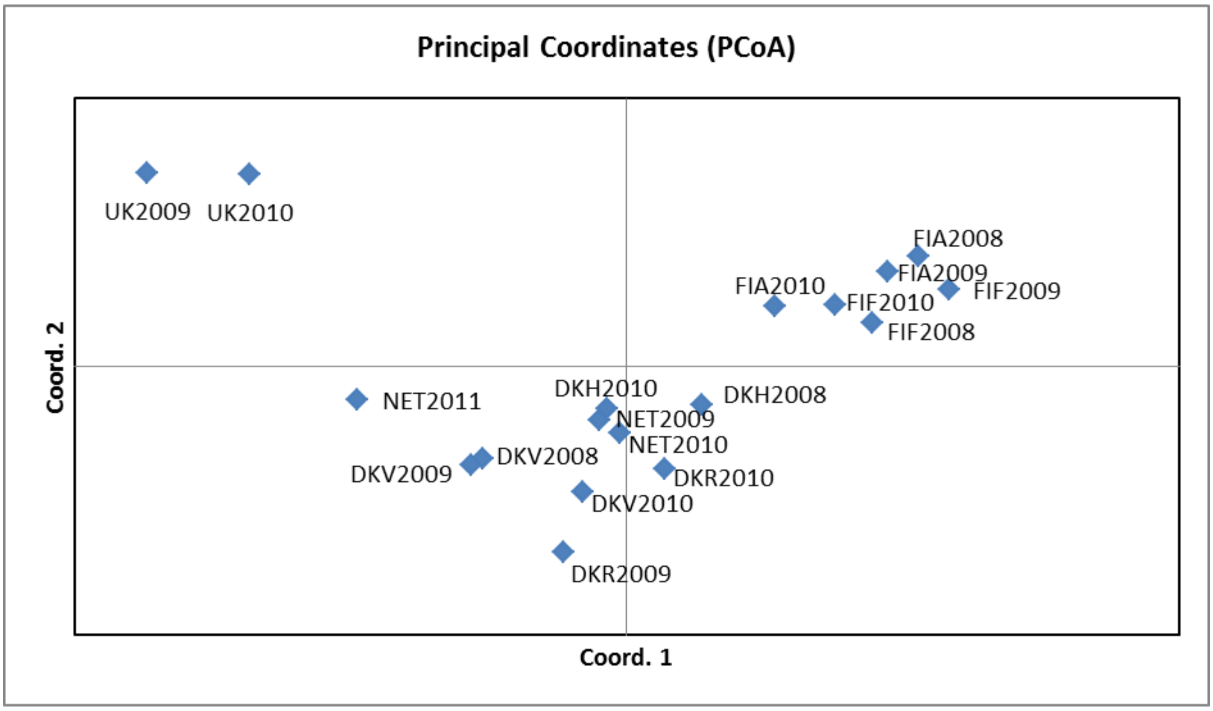

Supplement: Additional file 2 — Results of PCA analysis on linearized pair-wise FST. Differences among the temporal samples and populations are visualized. [file 1471-2148-14-12-S2.pdf]
