# Supplementary material for: Temporal genetic structure in a poecilogonous polychaete: the interplay of developmental mode and environmental stochasticity
Source: BMC Evol Biol. 2014 Jan 22;14:12. doi: 10.1186/1471-2148-14-12 (PMC3905951; doi:10.1186/1471-2148-14-12)
Supplement: Additional file 3 — Genetic variation in the temporal samples after the removal of some full-sib individuals. Observed (HO) and expected (HE) heterozygosity and inbreeding coefficients (FIS) are reported. [file 1471-2148-14-12-S3.pdf]

Additional file 3. Genetic variation in the temporal samples after the removal of full-sib individuals

Genetic variation in the temporal samples after the removal of full-sib individuals (all samples are included but samples in bold have full-sibs removed). Observed (H_O) and expected (H_E) heterozygosity, inbreeding coefficient (F_{IS}, with significant values underlined) are reported.

Sample	H_O	H_E	$F_{IS\ OLD}$	FIS no full-sibs
**FIA2008**	0.57896	0.65804	0.116	0.115
**FIA2009**	0.56272	0.66314	0.140	0.140
FIA2010	0.68795	0.71434	0.015	0.015
FIF2008	0.53108	0.64313	0.153	0.153
FIF2009	0.47757	0.61436	0.202	0.202
FIF2010	0.56730	0.65473	0.128	0.128
**DKR2009**	0.59758	0.69896	0.146	0.126
**DKR2010**	0.60993	0.68942	0.092	0.089
**DKV2008**	0.65147	0.76540	0.126	0.114
**DKV2009**	0.71428	0.77757	0.076	0.067
**DKV2010**	0.69450	0.72946	0.030	0.022
DKH2008	0.59607	0.67679	0.099	0.099
**DKH2010**	0.56731	0.72006	0.189	0.187
**NET2009**	0.58282	0.70370	0.169	0.173
**NET2010**	0.60589	0.68921	0.124	0.122
NET2011	0.61595	0.75168	0.182	0.182
UK2009	0.60609	0.79936	0.242	0.242
UK2010	0.66405	0.79743	0.165	0.165
