# Supplementary material for: Temporal genetic structure in a poecilogonous polychaete: the interplay of developmental mode and environmental stochasticity
Source: BMC Evol Biol. 2014 Jan 22;14:12. doi: 10.1186/1471-2148-14-12 (PMC3905951; doi:10.1186/1471-2148-14-12)
Supplement: Additional file 4 — Within population temporal pair-wise FST values after the removal of some full-sib individuals. [file 1471-2148-14-12-S4.pdf]

**Additional file 4. Within population temporal pair-wise $F_{ST}$ values after the removal of full-sibs individuals.**

Within population temporal pair-wise $F_{ST}$ values after the removal of full-sibs individuals (bolded temporal samples had full-sibs removed).

| **Finland** | | |
|---|---|---|
| FIA | **2008** | **2009** |
| **2009** | 0.001 | |
| 2010 | 0.008** | 0.004 |

| **Denmark** | |
|---|---|
| DKR | **2009** |
| **2010** | 0.003 |

| DKH | 2008 |
|---|---|
| **2010** | 0.007 |

| DKV | **2008** | **2009** |
|---|---|---|
| **2009** | 0.001 | |
| **2010** | 0.003 | 0.003 |

| **Netherlands** | | |
|---|---|---|
| NET | **2009** | **2010** |
| **2010** | 0.001 | |
| 2011 | 0.037*** | 0.032*** |

*P<0.05, **P<0.01, ***P<0.001
