# Supplementary material for: Temporal genetic structure in a poecilogonous polychaete: the interplay of developmental mode and environmental stochasticity
Source: BMC Evol Biol. 2014 Jan 22;14:12. doi: 10.1186/1471-2148-14-12 (PMC3905951; doi:10.1186/1471-2148-14-12)
Supplement: Additional file 5 — Mean relatedness (r) in the samples calculated with and without full-sibs (no FS). In DKR 2009, the mean relatedness is significantly lower after the removal of full-sib individuals. [file 1471-2148-14-12-S5.pdf]

Additional file 5. Mean relatedness (r) with and without full-sibs (no FS)

Mean relatedness (r) in the samples calculated with and without full-sibs (no FS). In DKR 2009 (in bold), the mean relatedness is significantly lower after the removal of full-sib individuals.
SE = standard error

Sample	Mean r (SE)
FIA 2008 / FIA 2008 (no FS)	0.0476 (0.002) / 0.0476 (0.002)
FIA 2009 / FIA 2009 (no FS)	0.0444 (0.001) / 0.0438 (0.002)
DKH 2010 / DKH 2010 (no FS)	0.0423 (0.002) / 0.0403 (0.002)
DKV 2008 / DKV 2008 (no FS)	0.0486 (0.002) / 0.0475 (0.002)
DKV 2009 / DKV 2009 (no FS)	0.0449 (0.002) / 0.0458 (0.002)
DKV 2010 / DKV 2010 (no FS)	0.0463 (0.003) / 0.0463 (0.002)
**DKR 2009 / DKR 2009 (no FS)**	0.0623 (0.003) / 0.0499 (0.003)
DKR 2010 / DKR 2010 (no FS)	0.0497 (0.003) / 0.0491 (0.002)
NET 2009 / NET 2009 (no FS)	0.0521 (0.002) / 0.0516 (0.002)
NET 2010 / NET 2010 (no FS)	0.0458 (0.002) / 0.0431 (0.002)
